# The Integrin-Ligand Interaction Regulates Adhesion and Migration through a Molecular Clutch

**DOI:** 10.1371/journal.pone.0040202

**Published:** 2012-07-06

**Authors:** Lingfeng Chen, Miguel Vicente-Manzanares, Laurent Potvin-Trottier, Paul W. Wiseman, Alan Rick Horwitz

**Affiliations:** 1 Department of Cell Biology, University of Virginia, Charlottesville, Virginia, United States of America; 2 School of Medicine at the Hospital de la Princesa, Universidad Autonoma de Madrid, Madrid, Spain; 3 Department of Physics, McGill University, Montreal, Quebec, Canada; 4 Department of Chemistry, McGill University, Montreal, Quebec, Canada; Leiden University, The Netherlands

## Abstract

Adhesive and migratory behavior can be cell type, integrin, and substrate dependent. We have compared integrin and substrate differences using three integrin receptors: α5β1, α6β1, and αLβ2 expressed in a common cell type, CHO.B2 cells, which lack integrin α subunits, as well as in different cell types that express one or more of these integrins. We find that CHO.B2 cells expressing either α6β1 or αLβ2 integrins migrate and protrude faster and are more directionally persistent on laminin or ICAM-1, respectively, than CHO.B2 cells expressing α5β1 on fibronectin. Despite rapid adhesion maturation and the presence of large adhesions in both the α6β1- and αLβ2-expressing cells, they display robust tyrosine phosphorylation. In addition, whereas myosin II regulates adhesion maturation and turnover, protrusion rates, and polarity in cells migrating on fibronectin, surprisingly, it does not have comparable effects in cells expressing α6β1 or αLβ2. This apparent difference in the integration of myosin II activity, adhesion, and migration arises from alterations in the ligand–integrin–actin linkage (molecular clutch). The elongated adhesions in the protrusions of the α6β1-expressing cells on laminin or the αLβ2-expressing cells on ICAM-1 display a novel, rapid retrograde flux of integrin; this was largely absent in the large adhesions in protrusions of α5β1-expressing cells on fibronectin. Furthermore, the force these adhesions exert on the substrate in protrusive regions is reduced compared to similar regions in α5-expressing cells, and the adhesion strength is reduced. This suggests that intracellular forces are not efficiently transferred from actomyosin to the substratum due to altered adhesion strength, that is, avidity, affinity, or the ligand-integrin-actin interaction. Finally, we show that the migration of fast migrating leukocytes on fibronectin or ICAM-1 is also largely independent of myosin II; however, their adhesions are small and do not show retrograde fluxing suggesting other intrinsic factors determine their migration differences.

## Introduction

Cell migration is a complex process comprised of multiple integrated and regulated steps [1], [2]. During migration, actin polymerization produces the forces that drive protrusion and retrograde flow of F-actin at the leading edge [3]–[5]. These forces are coupled to the substratum through integrin-based adhesions, which serve as traction points over which the cell moves as well as sources of migration-related regulatory signals [2], [6]–[8]. The efficiency of force transmission from the force generating systems in the cell to the substratum depends on the efficiency of a molecular clutch that connects adhesions to actin filaments [9], [10]. Myosin II has emerged as a critical regulator and integrator of cell migration [11]. By organizing the actomyosin cytoskeleton and generating contractile forces, it determines front-back polarity, regulates adhesion and the signals they produce, and mediates rear retraction. It also integrates the spatially separated processes that comprise migration and interprets the pliability of the substratum through a poorly understood signaling loop [11], [12].

While a picture of adhesion function and the pivotal role of myosin II and actin polymerization in cell migration are clear [2], [8], most of the data have been generated in fibroblasts adhering to fibronectin or vitronectin using either the α5β1 or αvβ3 integrins. However, other cell types, integrins and substrates have not been studied in comparable detail and may be different, since cells utilizing them have different migratory properties and adhesions. For example, many cells migrate on laminin, a process mediated mainly by the α6β1 integrin [13]. Also, leukocyte migration on ICAM-1 is characterized by high cellular speed, short and rapidly extending protrusions, and small, almost undetectable adhesions [14].

To investigate migration mechanisms using different integrins and substrates, we have used CHO.B2 cells, a CHO cell variant that expresses the integrin β1 subunit but almost no α subunit and therefore does not adhere or migrate on substrates like fibronectin [15], [16]. We expressed the α5 or α6 integrin subunit in CHO.B2 cells and measured their migration on fibronectin and laminin, respectively. We also transfected a leukocyte-specific integrin, αLβ2, into CHO.B2 cells and measured their migration on ICAM-1, an inflammation-related substrate [17]. To parse contributions arising from integrin-substrate interactions and cell type, we investigated cell types that naturally express the integrins studied in the CHO model. For α6β1 we used the osteosarcoma cell U2OS, which expresses both α5β1 and α6β1 [18] and therefore migrates on both fibronectin and laminin. For αLβ2, we used HL60 cells, which migrate robustly on ICAM-1. We found that myosin II plays a greatly reduced role in adhesion and migration of cells on laminin and ICAM-1 compared to that on fibronectin. This difference appears to arise from a novel retrograde fluxing of α6β1 and αLβ2 integrins that results in reduced adhesion strength and force transmission to adhesions. Leukocyte migration (using the HL-60 cells) was also largely myosin II-independent; but the differences in morphology of the cells and their adhesions from that of the CHO cells using the same integrins seem to reflect intrinsic cell type differences in actin organization.

## Materials and Methods

### Plasmids

α5-GFP has been described previously [16]; α6-GFP was made by excising the α6 cDNA from the α6-pRSVneo plasmid [19] and inserting into the 5′ of the pEGFP-N1 vector (Clontech) using the flanking Apa I and Kpn I sites; αL-GFP and β2-GFP were generated by in-frame replacement of YFP with GFP in αL-YFP and β2-YFP, which were kind gifts from T. Springer [17]. The low expression, “speckle” versions of α5-GFP, α6-GFP and αL-GFP were made by replacement of the CMV promoter with a truncated version that enables very low expression [20]. mCherry-MIIA and mCherry-MIIB were made from their GFP version as described [21], which were gifts from R. Adelstein [22]; wild type RLC-mCherry, and its mutant versions: RLC-AA-mCherry, RLC-AD-mCherry, RLC-DA-mCherry, RLC-DD-mCherry were prepared from RLC-GFP and RLC-DD (from Kathleen Kelly, NCI/NIH) as described previously [23], [24]. The mCherry plasmid was a gift from R. Tsien [25]. Paxillin-GFP and Paxillin-mCherry were also described previously [16], [26], [27]. Low expression, “speckle” mGFP-dSH2 and mCherry-dSH2 were generated from the YFP-SH2 construct donated by Benjamin Geiger [28] by replacement of the YFP with mGFP or mCherry and of the CMV promoter with a truncated version that enables very low expression [20]. Use of the mGFP version has been recently described [12].

### Cell Culture and Transfection

CHO.B2 cells (from Rudi Juliano) [15] and HL60 cells (from Orion Weiner) [29] were cultured in DMEM medium and RPMI medium 1640, respectively, from Invitrogen. CHO.B2 cells were transfected using Lipofectamine or Lipofectamine 2000 (Invitrogen) according to the manufacturer’s instructions. HL60 cells were differentiated and transfected with the Amaxa nucleofection system from Lonza [30].

U2OS cells and HT1080 cells were obtained from ATCC and transfected using Lipofectamine 2000 according to the manufacturer’s instructions.

### Antibodies and Reagents

Fibronectin and laminin were from Sigma-Aldrich. Recombinant human ICAM-1 (Fc fragment) was from R&D Systems. Phospho-RLC (pRLC) and total RLC antibodies were from Rockland Inc. and Sigma, respectively. MIIA and MIIB antibodies were from Covance.

### Immunoblots

Cells (∼10^6^) were incubated on the indicated substrates for one hour, washed using PBS (Invitrogen), and lysed in RIPA buffer (Pierce). The resulting lysates were separated by 4–20% SDS/PAGE (BioRad). Proteins were transferred onto PVDF membranes, blocked using SuperBlock blocking buffer (Thermo Scientific) and immunoassayed for pRLC, MIIA, MIIB or total RLC by Western blot using Amersham ECL system (GE healthcare). When indicated, densities were quantified using ImageJ.

### Migration Assays

Cell migration was assayed under 10X phase microscopy (Nikon TE300). The cells were plated in CMM1 medium (Hyclone from Thermo Scientific), and allowed to migrate for the desired time. Images were captured using a CCD camera (Hamamatsu) with Metamorph software (Molecular Devices) and then analyzed by ImageJ as previously described [31].

### TIRF Microscopy

TIRF images were acquired on an Olympus IX70 inverted microscope (1.45 NA (oil) PlanApo 60X TIRFM objective), fitted with a Ludl modular automation controller (Ludl Electronic Products) and controlled by Metamorph (Molecular Devices). GFP and mCherry were excited using the 488 nm laser line of an Argon ion laser and the 543 nm laser line of a He-Ne laser (Melles Griot), respectively. A dichroic mirror (HQ485/30) was used for GFP-labeled cells. For dual GFP- mCherry/mOrange, a dual emission filter (z488/543) was used. Images were acquired with a charge-coupled device (CCD) camera (Retiga Exi; Qimaging) controlled by Metamorph software.

To confirm the co-localization of adhesion components, some images were acquired using Olympus inverted microscope IX71 (1.45 NA (oil) PlanApo 100X TIRFM objective) fitted with a Dual-View (Photometrics) to simultaneously acquire both colors.

When indicated, substrates were covalently cross-linked to the coverslip using GMBS as previously reported [32]. For other experiments, we adsorbed the substrates to coverslips pre-coated with 1 mg/mL poly-L-lysine, which has been described to improve adsorption [33], [34].

All images were analyzed using ImageJ. For kymography, a single line is drawn from the edge of the protrusion toward the cell center, using images captured every 2 seconds, then the line intensity is plotted with the x-axis representing total time (4 minutes), and y-axis representing the movement of the cell edge [35], [36].

### Image Correlation Spectroscopy

STICS (spatio-temporal image correlation spectroscopy) [37] was used to quantify (magnitude and direction) transport of adhesion components during fluxing. This method measures the peak displacement of the spatio-temporal correlation function calculated from fluorescence intensity fluctuations recorded in a time series of images in order to compute the average velocity of the labeled species in a small region of interest. It was used previously in the same context to characterize the relative transport of different adhesion components [9]. The average velocities were computed from only the top 20% of measured velocities to remove non-fluxing vectors in the region of analysis. The total average was then computed over all cells (one region per cell).

Spatial image correlation spectroscopy (ICS) [38] was used to measure the relative density (expression level) of fluorescent proteins. Spatial correlation functions are calculated for each region and the amplitude of the peak, after background noise correction, is inversely proportional to the fluorescent protein density per focal spot area. One uniform region was selected per cell, and the time average was obtained for each cell analyzed with STICS.

### FACS

Cell sorting was done on a Becton Dickinson FACSVantage SE Turbo Sorter with DIVA Option at the Flow Cytometry Core Facility of UVA. A total of 2.0×10^7^ CHO.B2 cells were transfected and suspended in Basic Sorting Buffer (1x Ca/Mg^2+^ free PBS, 1 mM EDTA, 25 mM HEPES pH 7.0, 1% Heat-Inactivated FBS, filter sterile), then sorted into low, medium-low or high fluorescence groups, with at least 1×10^6^ cells for each condition. Cells were allowed to recover overnight in DMEM medium with 20% serum before analysis.

### FRAP and Bead Displacement

Confocal images for FRAP (fluorescence recovery after photobleaching) analysis and bead displacements were acquired using an Olympus inverted microscope IX81 (1.40 NA (oil) PlanApo 60X objective) driven by FluoView software (Olympus). Bead displacements were measured using red fluorescent (580/605) FluoSpheres® carboxylate-modified microspheres (Invitrogen) imbedded into polyacrylamide gels at ∼1 kPa stiffness [39], [40] coated with the specified substratum [41], [42]. CHO.B2 cells were transfected with the appropriate GFP integrin and 2-channel (GFP/beads) image time series of protrusion and retraction events were recorded. Bead displacements were determined using a particle tracking routine written in MATLAB (MathWorks) and interpolated onto a regular grid comprised of 32×32 pixels subregions to find the gel displacement. The relative gel deformations were referenced to the beginning of the protrusions (or end of retraction) so that the measured gel deformation was caused by force differences during those events.

For FRAP, a selected cellular area that contained GFP fusion protein was scanned five times, and then bleached using 15 scans at 100% laser power. To image the fluorescence recovery of fluorescence intensity after the photo-bleaching, we collected 200 scans in succession; 100 scans every 0.2 s followed by 50 scans every 0.5 s. Background subtraction and normalization were calculated for the averaged intensities from the bleach region, and normalized intensity vs. time were fitted by a single exponential equation. Data collected were processed using ImageJ, Excel (Microsoft) and SigmaPlot (SYSTAT) software.

### Adhesion Assay

Affinities of different integrins were measured using a centrifugation assay [43]. Cells were transfected with α5, α6 or αL+ β2 construct, placed into in 96-well plates coated with FN, LN or ICAM-1, respectively, and incubated for 20 minutes at 37°C in a CO_2_ incubator. The plates were then sealed, inverted and centrifuged at 200 rpm three times for 5 minutes using the Beckman GH 3.8 rotor. Control plates were sealed and inverted for 15 min but not centrifuged. Cells in each well were counted and compared to its positive well. The adhesion strength for each integrin was presented as the fraction of cells remaining after centrifugation.

## Results

### Integrin Heterodimer Expression Dictates Migration, Protrusion and Adhesion

**Figure 1:**
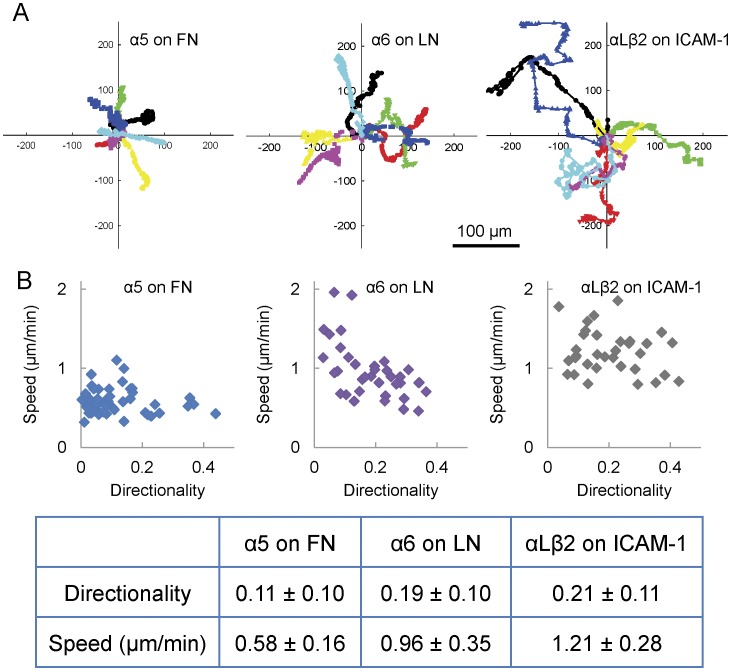
Migration of CHO.B2 cells expressing different integrins. (A) CHO.B2 cells expressing α5-GFP, α6-GFP, or αL-GFP + β2-GFP migrate randomly on fibronectin (FN), laminin (LN) or ICAM-1, respectively. Typical cell paths are shown, with each individual cell track assigned a different colored line translated to a common origin. Experimental time: 6 hours. Scale Bar  = 100 µm. (B) The speed and directionality, from at least three independent experiments, were calculated and plotted (cell number  = 52, 36, 33, respectively). The speed was calculated from the total length of a cell path divided by the experimental time. Cells on FN migrate about half as fast as cells on either LN or ICAM-1 (P<1×10^−9^), and the difference between cells on LN and ICAM-1 is small but still significant (P = 0.0017). Directionality was defined as the ratio of the length from the start to the end point and the length of the cell path.

To determine whether the migratory properties of cells depend on the integrin-ligand pairs utilized, we expressed either the α5 or α6 subunits or co-expressed the αL and β2 integrin subunits in CHO.B2 cells, a CHO cell variant that expresses the integrin β1 subunit but very little alpha subunit [15], and plated them onto fibronectin, laminin or an ICAM-1-Fc construct [44] (R&D Systems), respectively. α5-GFP expression mediated adhesion, spreading and migration on fibronectin but not on laminin or ICAM-1; α6-GFP promoted adhesion and spreading on laminin, but not on fibronectin or ICAM-1; simultaneous expression of αL-GFP and β2-GFP enabled adhesion and migration on ICAM-1 but not on fibronectin or laminin, and CHO.B2 cells expressing no ectopic integrin neither spread nor migrated on any substrate tested under comparable conditions (Figure S1, data not shown). Figure 1A shows the paths of seven typical cells expressing comparable levels of integrin-GFP for each condition plotted from a common origin traced over identical time periods. The cells on ICAM-1 migrate the fastest (larger displacement per unit time) and are the most directionally persistent (total net displacement from the origin), with cells on laminin intermediate and those on fibronectin slowest and least directionally persistent. The average speed of cells on fibronectin is about half that of cells on either laminin or ICAM-1 (Figure 1B, lower panel, P<1×10^−9^). While the difference in speed between cells migrating on laminin and ICAM-1 is not as large, it is still significant (P  = 0.0017). The differences in directional persistence were estimated as the ratio of the distance between the start and end points of the migration path to the total length of the path. The directionality of cells on fibronectin was much lower than that of cells on either laminin or ICAM-1 (P value of fibronectin vs. laminin  = 0.00078); whereas the cells on fibronectin had significant movement but little net translation (Figure 1B, upper panel).

**Figure 2:**
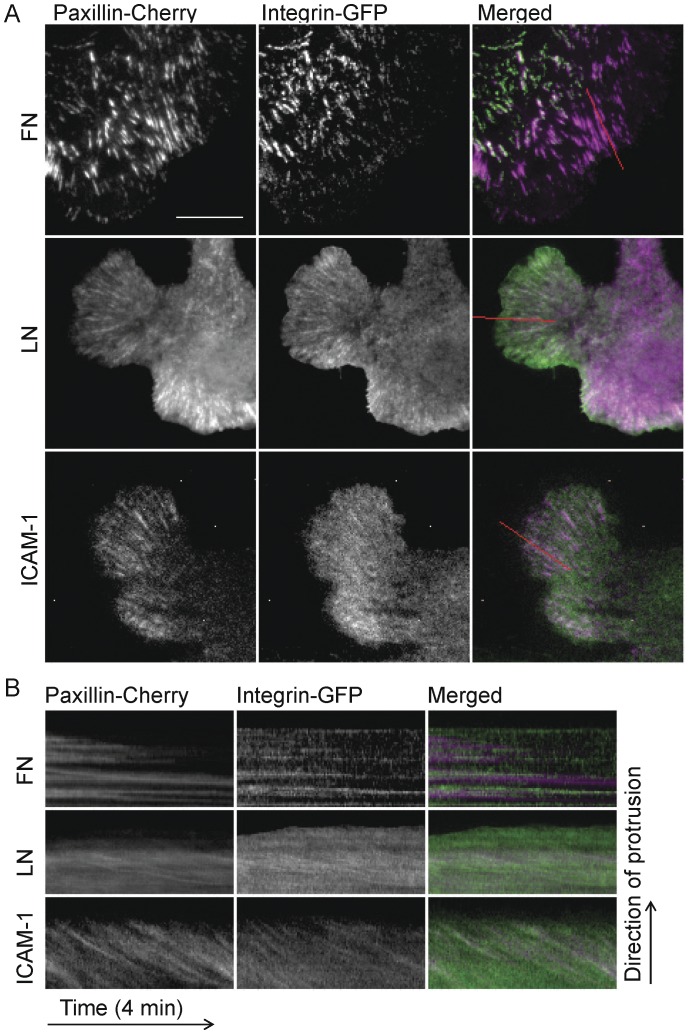
CHO.B2 cells expressing different integrins show differences in protrusion and adhesion. (A) CHO B2 cells were co-transfected with paxillin-mCherry and α5-GFP, α6-GFP or αL-GFP + β2-GFP and then plated on FN, LN or ICAM-1. In the merged color panels, paxillin is in magenta and integrins are in green. Scale Bar  = 10 µm. (B) Kymographs of cell edge and adhesions in protrusions. The retrograde fluxing of the integrins and paxillin on LN or ICAM-1 are revealed by the movement of discrete molecular units within the adhesion; this is apparent in the downward parallel line formed in the kymographs that overlie adhesions: paxillin (left), integrin (center) and merged (right). Note that the entire adhesion remains largely in place during the fluxing on LN; this also occurs on ICAM-1.

We also analyzed the protrusions and adhesions of cells expressing α5-, α6-, or αLβ2-GFP and paxillin-mCherry migrating on fibronectin, laminin or ICAM-1, respectively. Cells migrating on laminin or ICAM-1 exhibited more numerous and rapid protrusions, as quantified by kymography, than those on fibronectin (Movie S1). Cells on laminin or ICAM-1 extended and retracted their protrusions more rapidly and frequently (∼2.3±0.5 µm/min) than cells on fibronectin (1.6±0.5 µm/min, P<<0.001; Movie S1). This correlates well with the observed differences in migration. Adhesions, as visualized using paxillin-mCherry, were also different. Cells on fibronectin displayed prominent small, dynamic, nascent adhesions that actively turned over at the front of protrusions, as well as some larger, more stable, and slightly elongated adhesions in the more distal portions of protrusions (Figure 2A) [26], [45]. In contrast, cells spread on laminin or ICAM-1 had few nascent adhesions; most adhesions in protrusions assembled and elongated quickly, and therefore, were highly elongated from very early time points (Figure 2A).

To determine whether these observations are cell type-independent, we investigated the adhesion and migration of U2OS and HT1080 cells. These cells adhere to fibronectin through α5β1; but unlike CHO cells, they also spread and migrate spontaneously on laminin, likely due to their endogenous α3β1 and/or α6β1 [46]–[48]. Both the U2OS and HT1080 showed higher protrusiveness on laminin than on fibronectin (Figure S2). In addition, adhesions in cells on laminin elongated quickly (data not shown). Adhesion maturation was also observed in cells on fibronectin (data not shown). These results are similar to those presented above for the CHO cells and suggest that the observed behaviors on different substrates are due to intrinsic differences in the integrin-ECM interactions rather than different integrin expression levels or cell type-dependent differences.

### The Differences among Cells Migrating on Fibronectin, Laminin and ICAM-1 do not Originate from Major Alterations in Myosin II Activity

**Figure 3:**
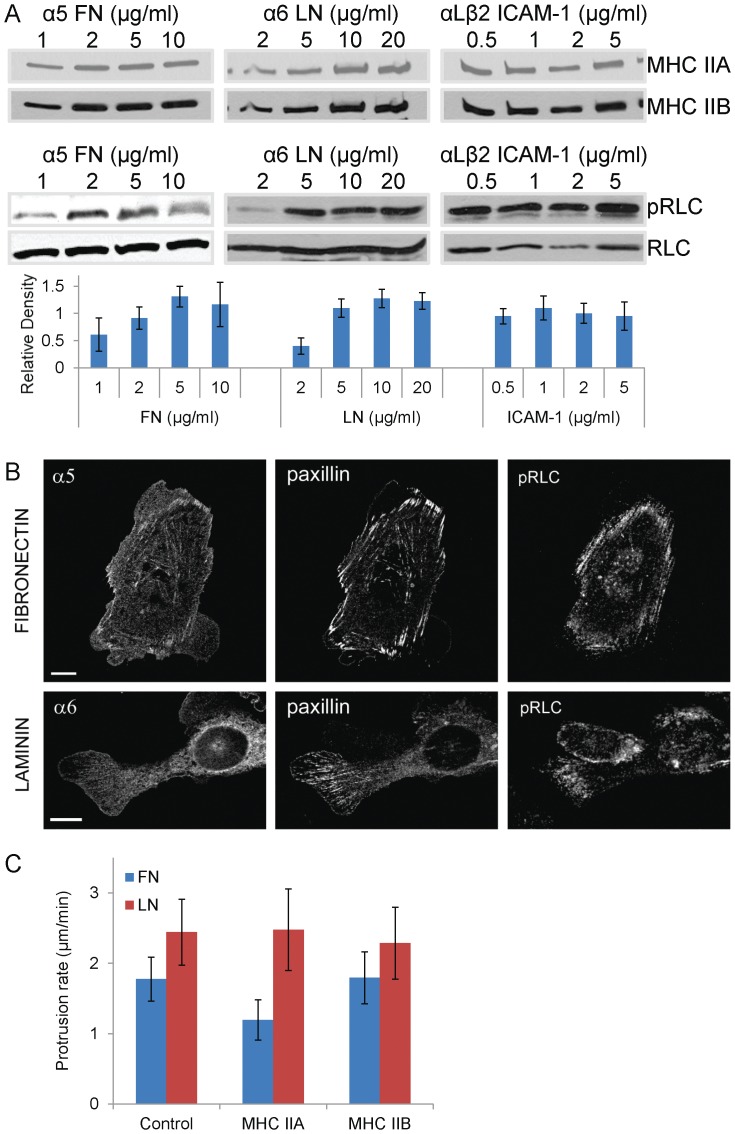
Effect of myosin II on protrusion. (A) CHO.B2 cells transfected with the indicated fluorescently tagged integrin subunit(s) were platted on the indicated substrate for 1 hour, then blotted for MHC IIA, MHC IIB, phosphorylated (p) and total RLC. pRLC does not increase noticeably on ICAM-1 but does on both FN and LN in a dose dependent manner. Note also that MHC IIA and MHC IIB do not change with substrate concentration. Also, the densitometric quantification of pRLC corrected for load using total RLC is shown under each blot. At least three experiments were quantified for each substrate. (B) Adhesion on fibronectin (α5β1) or laminin (α6β1) determines the subcellular distribution of pRLC (Ser19). CHO.B2 cells were (top) transfected with α5-GFP and plated for 60 min on FN (2 µg/ml); (bottom) CHO B2 cells transfected with α6-GFP and plated for 60 min on LN (10 µg/ml). The cells were stained for paxillin and phosphorylated (Ser19) RLC as indicated. Note the more fibrillar distribution of the pRLC in the cells on FN. Scale bar = 10 µm. (C) Over-expression of MHC IIA, but not MHC IIB, inhibits protrusion of CHO.B2 cells on FN but not on LN. CHO.B2 cells were doubly transfected with α5- or α6-GFP and mCherry-MHC IIA or MHC IIB as indicated, then plated on the corresponding substrate (FN for α5, LN for α6). Scale Bar  = 10 µm. Protrusion rates from 4 minute movies were calculated from kymographs and plotted. Data are expressed as the mean ± SD of at least 3 independent experiments. (Protrusion number  = 7, 7, 12, 12, 10, 11, respectively.) P<0.001 for cells on FN expressing ectopic MHC IIA compared to cells expressing ectopic MHC IIB or control cells.

Myosin II plays a pivotal role in the adhesion and migration of cells on fibronectin [49], [50], [21], [23], [12], and therefore, we asked whether the integrin heterodimer-dependent changes in migration and adhesion arise from differences in myosin activation or isoform expression. We first examined myosin II activation in CHO.B2 cells transfected with α5, α6, or αL and β2 that were plated onto fibronectin, laminin or ICAM-1, respectively. Myosin II activation was assessed by immunoblotting for phosphorylated RLC. CHO.B2 cells expressing the α5β1 or α6β1 integrins and plated on fibronectin or laminin, respectively, showed a substrate concentration-dependent RLC phosphorylation (Figure 3A). However, the distribution of phosphoRLC (pRLC) was markedly different. In α5-expressing cells on fibronectin, pRLC localized robustly along thick actomyosin fibers that terminate in large adhesions. In α6-expressing cells on laminin, pRLC localized to thinner, strand-like actin structures in protrusions (Figure 3B). In contrast, RLC phosphorylation was low and largely substrate concentration-independent in αLβ2-expressing CHO.B2 on ICAM-1. CHO.B2 cells expressing α5-, α6- or αL-+β2-GFP migrating on fibronectin, laminin, or ICAM-1, respectively, showed no significant difference in the relative expression of MHC IIA, MHC IIB, or RLC (Figure 3A). Taken together, these data do not reveal a tight correlation between substrate/integrin utilization, migration and RLC phosphorylation for all integrins tested.

To investigate further whether the differences observed in adhesion and migration were due to the differential activation or other effect of myosin II on cells expressing the different integrin/ligand pairs, we perturbed myosin II expression and activity in cells on different substrates. Overexpression of MHC IIA, but not MHC IIB, decreased the protrusion rates of α5 expressing CHO.B2 cells (Figure 3C), as previously shown in CHO.K1 cells [21]. However, overexpression of MHC IIA did not affect protrusion in the α6β1-expressing CHO.B2 cells (Figure 3C) and in αLβ2-expressing cells (data not shown). Similarly, myosin II activation by overexpression of phospho-mimetic RLC mutants (RLC-A,D and RLC-D,D) [23], [24], or inhibition by addition of ML7 and Y27632 to inhibit MLCK and ROCK, respectively, which are upstream of RLC phosphorylation in these cells [23], [24], did not show significant differences on protrusion rates (Figure S3). Thus, enhancing myosin II expression or activity has little effect on protrusion and adhesion in cells using α6β1 or αLβ2 for migration, and therefore, differences in migration properties between CHO.B2 cells with α6β1 or αLβ2 integrins do not appear to result primarily from alterations in myosin II activity.

To ensure that possible variations in integrin expression do not produce the observed phenotypes, we used Fluorescence-Activated Cell Sorting (FACS) to sort cells by the expression level of integrin-GFP in CHO.B2 cells. The cells were binned into three populations: low, medium-low, and high fluorescence; this range of expression includes the endogenous level in wild type cells. Immunoblotting and kymography were performed on the three populations. Although pRLC levels increased somewhat in the cells expressing high α5-GFP, the protrusion rates remained similar for each integrin expressed (Figure S4A). This result suggests that the observed behaviors on different substrates are due primarily to intrinsic differences in the integrin-ECM binding rather than differences in integrin expression levels. Similar results were observed in cells expressing α6-GFP or αL-GFP (Figure S4A, data not shown).

### The Integrin-ligand Interactions of the α6β1 and αLβ2 Integrins Differ from those of α5β1

The absence of a clear relation between myosin II activity and migration, adhesion and protrusion on the different substrates suggests that the myosin II activity is not coupled efficiently to signaling and adhesion in cells using some integrins. Therefore, we queried whether there are differences in the efficiency of the linkage, or molecular clutch, that couples actin and adhesion [9], [51], [52]. To do this, we measured the retrograde movement, or fluxing, of adhesion components in protrusions, since these adhesions are the traction points through which cellular forces are shunted to the substratum and thereby inhibit retrograde flow. Interestingly, in cells expressing either α6 or αLβ2, most of the elongated adhesions in protrusions exhibited a rapid retrograde, flux of paxillin, which is seen as parallel downhill slopes in the kymographs (Figure 2B). Conversely, in cells on fibronectin, we did not observe robust centripetal fluxing of paxillin in adhesions 26], except in adhesions located within regions that were retracting actively.

**Figure 4:**
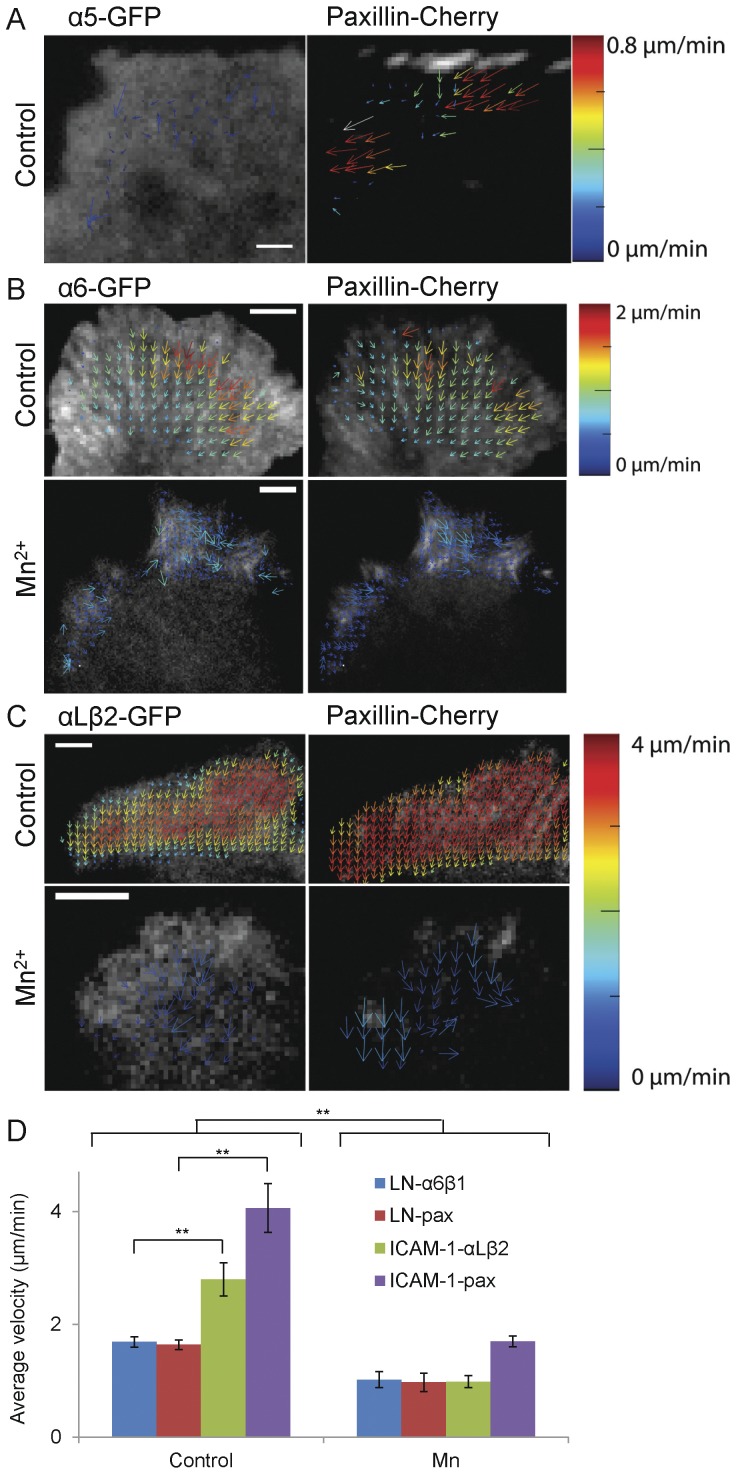
STICS measurements of the retrograde fluxing of paxillin and integrin in CHO.B2 cells plated on FN, LN and ICAM-1 with or without Mn^2+^. Cells were doubly transfected with the appropriate integrin (left) and paxillin-mCherry (right). (A) α5-expressing cells seldom show slow retrograde flux of paxillin or integrin in adhesions in protruding regions. (B) α6β1 fluxes retrograde in protrusions and is inhibited from 1.68±0.09 to 1.0±0.1 µm/min (P = 0.0051) by addition of Mn^2+^. Paxillin fluxes are inhibited from 1.64±0.09 to 1.0±0.2 µm/min (P = 0.0051) (C) αLβ2 fluxes retrograde fluxing in protrusions is inhibited by almost a factor of 3 (from 2.8±0.3 to 1.0±0.1 µm/min, P = 0.0016) and that for paxillin are inhibited from 4.1±0.4 to 1.7±0.1 µm/min (P = 0.0016). Scale Bar  = 5 µm. Protein velocity is represented using the rainbow color scale bar. (D). Average fluxing velocity for each condition is presented as the mean ± SEM. αL fluxes faster than α6, along with paxillin (P = 0.0025). The results are from analyses of 29 cells.

**Figure 5:**
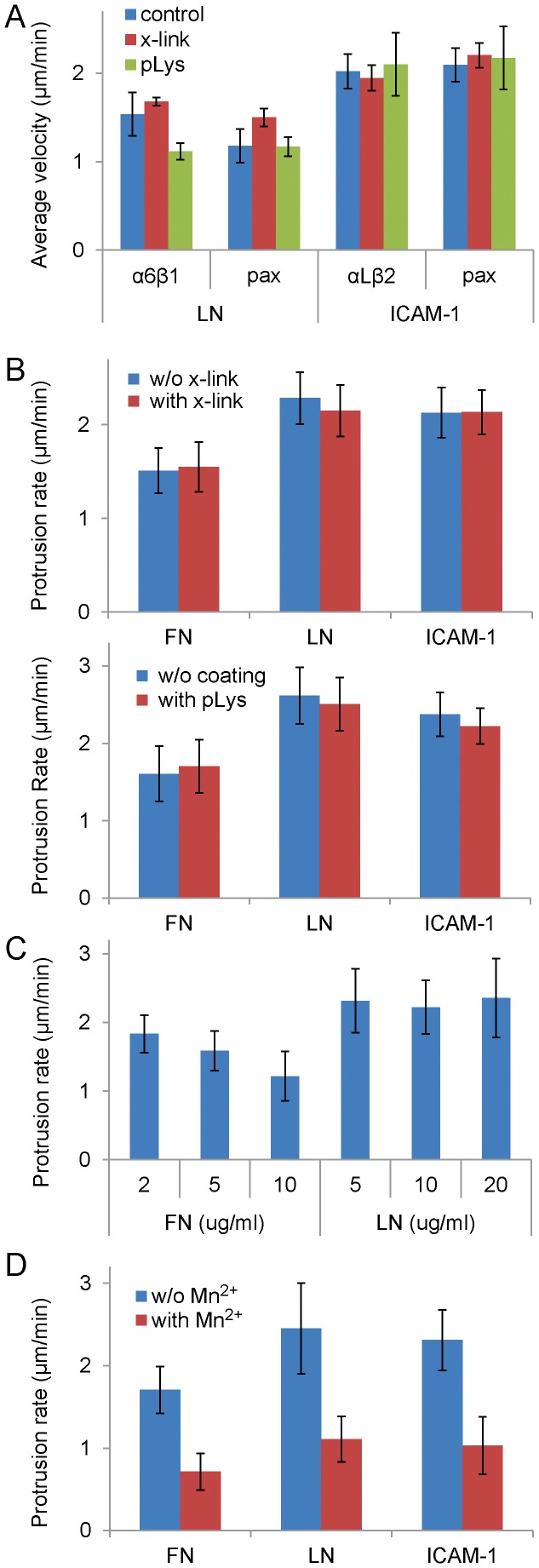
The efficiency of the ECM-actin linkage differs between α5β1 and α6β1 or αLβ2. CHO.B2 cells were doubly transfected with paxillin-Cherry and the indicated integrin (with GFP), then either plated on different concentration of substrates or cross-linked or poly-lysine treated substrates or treated with Mn^2+^. (A) Fluxing is not affected by covalent cross-linking (x-link) or poly-L-lysine attachment (pLys). CHO-B2 cells doubly transfected with paxillin-Cherry and the indicated integrin (with GFP), then either plated on control, cross-linked or poly-L-lysine attached substrates. Average velocity of protein fluxing was quantified by STICS. Data are expressed as the mean ± SEM (n = 28 cells). Two-way ANOVA reveals that neither poly-L lysine attachment nor crosslinking have a significant effect on the flux velocity (P = 0.48). (B) Covalent cross-linking (Upper) or poly-L-lysine attachment (Lower) of the indicated integrin ligands did not significantly affect protrusion. Data are expressed as the mean ± SD of at least 3 independent experiments (n = 7, 7, 12, 15, 10, 7 for cross-linking, respectively; n = 8, 9, 8, 9, 8, 7 protrusions for poly-lysine, respectively.) Protrusion rates were obtained from kymographs. (C) Protrusion rates of cells expressing α5 or α6 integrins and plated on FN or LN, respectively. On FN, the protrusion rate decreases with increasing concentration (P<0.006). No significant difference was detected with LN. (D) Protrusion of CHO.B2 cells was inhibited by activating integrins with Mn^2+^ (P<1×10^−6^). Data are expressed as the mean ± SD of at least 3 independent experiments. (n = 9, 9, 10, 12, 9, 10 protrusions respectively.).

Previous studies have identified a “slip” point within the adhesion-actin linkage. That is, in some adhesions, the integrins remain fixed while other adhesion molecules flux; α-actinin, which is bound to actin, moves the fastest [9], [10]. To localize the “slip” point, in the linkage with integrins, we assayed the movement of adhesion components including the α6 and αL integrins on laminin and ICAM-1 using their GFP derivatives at expression levels low enough to observe “speckles” [53]. Surprisingly, all of the components (data not shown) including the integrins displayed robust retrograde movement even when the protrusions were stationary; however a comparable, retrograde fluxing of α5 in cells on fibronectin was seldom seen (Figure 4) [9], [51]. The integrin fluxing does not appear to arise from a weak interaction between the matrix ligand and the glass coverslip, since laminin covalently cross-linked to the coverslip or adsorbed onto pre-bound poly-L-lysine, which improves laminin binding [33], [34], did not have a significant effect on the flux and protrusion rates of α6 or paxillin (Figure 5A, B). In addition variations in integrin expression levels do not correlate well with the level of fluxing (Figure S4B). Also, unlike cells expressing α5 on fibronectin, the protrusion rate of α6 or αLβ2 does not appear to arise from the density of laminin (Figure 5C) or ICAM-1 (data not shown). While few cells attached to very low concentrations of ICAM-1, those that did went on to migrate (data not shown). In contrast, increasing the density of fibronectin decreased the protrusion rate; however, lowering the fibronectin concentration to the threshold for adhesion of these cells, 0.5–1 µg/ml, did not increase protrusion to a rate comparable to that seen for cells on LM or ICAM-1 (data not shown). These observations further suggest that the differences among different integrin-ligand pairs are intrinsic.

Finally, we altered the strength of the integrin-substrate interaction by adding Mn^2+^ [54], which increases integrin affinity, to CHO.B2 cells expressing paxillin-Cherry and α6- or αLβ2-GFP. Mn^2+^ inhibited the retrograde fluxing and the protrusion rates of both α6 and αLβ2 (Figure 4D, Figure 5D). We quantified these differences using spatio-temporal image correlation spectroscopy (STICS) [37], [9]. In the presence of Mn^2+^ the rate of retrograde flux for αLβ2 decreased ∼3-fold in cells on ICAM-1 (Figure 4C, from 2.8±0.3 to 1.0±0.1 µm/min, P = 0.0016); while the rate of paxillin slowed from 4.1±0.4 to 1.7±0.1 µm/min (P = 0.0016). A similar effect was observed with α6-expressing cells migrating on laminin (Figure 4B); the average retrograde flux of α6 slowed from 1.68±0.09 to 1.0±0.1 µm/min (P = 0.0051), which is similar to that of paxillin (from 1.64±0.09 to 1.0±0.2 µm/min, P = 0.0051). In the absence of Mn^2+^, cells plated on laminin exhibited faster flux for both proteins than those plated on fibronectin (P<0.01).

**Figure 6:**
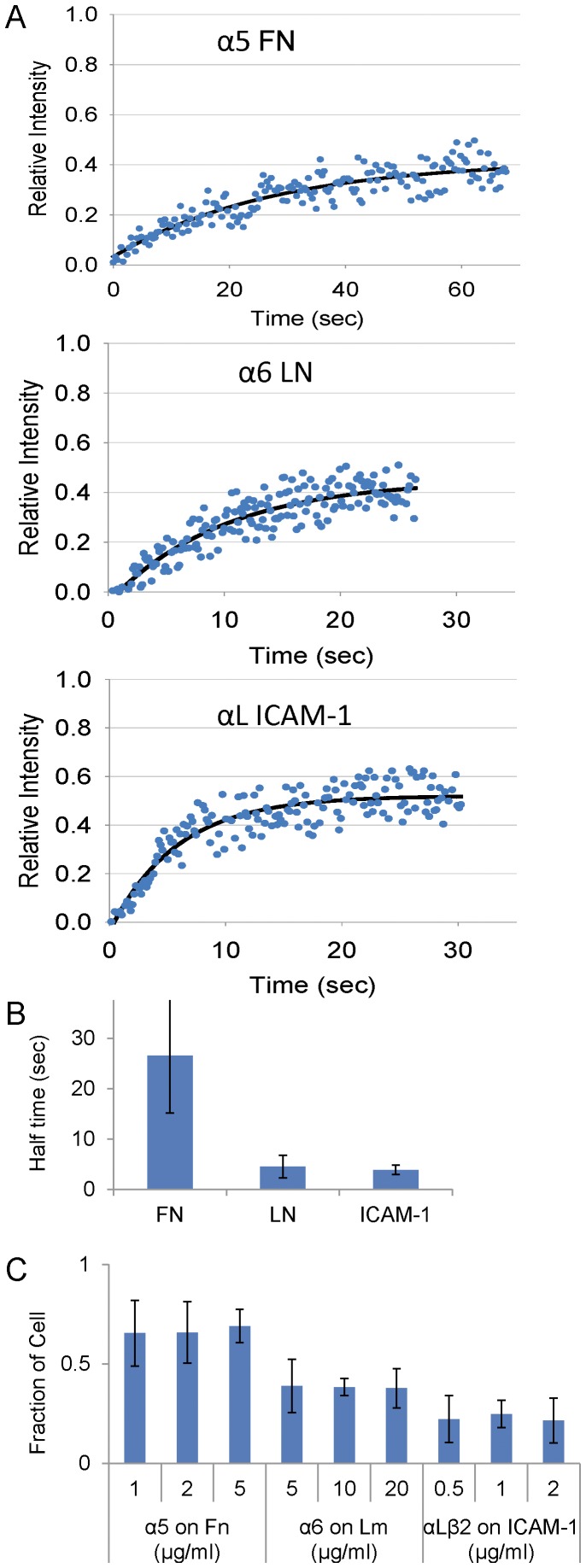
Fluorescence Recovery After Photobleaching (FRAP) and adhesion assays for CHO.B2 cells expressing α5, α6 or αL+β2 integrins and migrating on FN, LN and ICAM-1, respectively. (A) Typical FRAP curve for each condition. Data points are in blue, and a single exponential fit is in black. Notice the time scale differences. The R^2^ for a single and double exponential fit were both 0.8. From the single exponential fits, the fractional recoveries for the three typical curves are 0.39, 0.49, 0.56, respectively; estimated half-times (T_1/2_, sec) are 19, 6.3, 4.1, respectively. (B) From the single exponential fits, recovery half-times (T_1/2_) were plotted as mean ± SD. The T_1/2_ for α5-GFP is significantly larger than for either α6-GFP or αLβ2-GFP (n = 3 cells for each condition, P<0.05). (C) Adhesion strength assay. Cells expressing α5, α6 or αL+β2 integrins were plated onto substrates coated with FN, LN, or ICAM-1, then either simply inverted (positive control), or inverted and centrifuged at low speed (200 RPM). The number of remaining cells was counted. The relative adhesion strength was estimated by the fraction of cells remained after centrifugation divided by the positive control. More cells remained on FN than on LN or ICAM-1: P<0.04.

The dramatic and novel differences in retrograde integrin fluxing suggest that the adhesion strength, i.e., the apparent affinity, avidity or ligand-integrin-actin linkage, of α5β1 for fibronectin is higher than that for either α6β1or αLβ2 interacting with its respective ligand. To test this, we used fluorescence recovery after photobleaching (FRAP). This measures the diffusion of the integrins in the membrane plane, a parameter that would be affected by the affinity of integrin for its ligand and alterations in the integrin-actin linkage. CHO.B2 cells expressing α5-GFP, α6-GFP or αLβ2-GFP were plated on fibronectin, laminin or ICAM-1, respectively, and the mobility of the fluorescently-labelled integrin was measured by FRAP. The data in Figure 6A, B shows that the recovery of α6 or αLβ2 after photobleaching is significantly faster, and the fractional recovery higher, than that of α5. Since diffusion in the membrane is largely insensitive to the size of the integrin [55], [56], the data support an altered ligand-integrin-actin linkage that is stronger between α5 and fibronectin than between α6 and laminin, or αLβ2 and ICAM-1.

Finally, we measured the adhesion strength directly using a centrifugation assay. In this assay, cells are plated and allowed to adhere, and then the plates are inverted and centrifuged [43]. The fraction of cells remaining on the dish is a measure of the relative adhesion strength. The fraction of cells on remaining on fibronectin is significantly higher than that on laminin or ICAM-1 (P<0.04) (Figure 6C). This shows that the adhesion strength of α5β1 to fibronectin is stronger than that of α6β1 to laminin or αLβ2 to ICAM-1 under these conditions.

### α6β1 Adhesions Show Reduced Traction Forces but Still Signal

**Figure 7:**
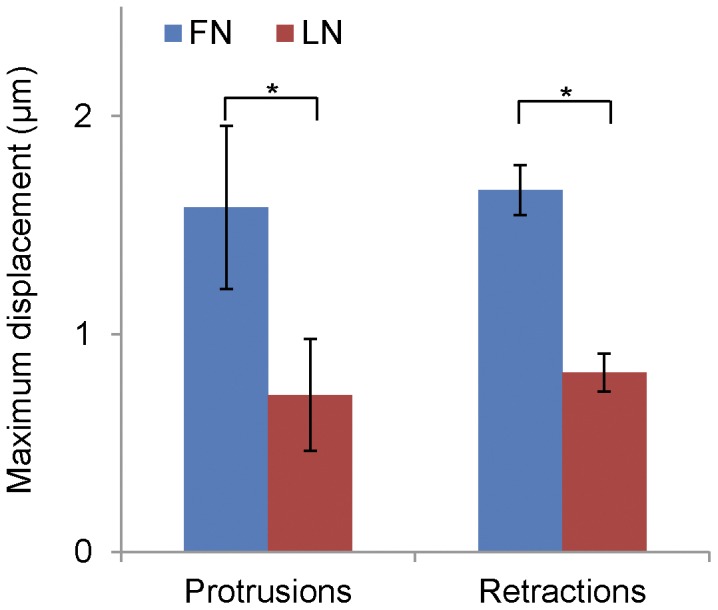
Bead displacements for CHO.B2 cells migrating on FN or LN. Cells were plated onto bead embedded polyacrylamide substrates (E = 1 kPa) coated with adhesive ligand. The relative bead displacements were measured in regions of protrusion, retraction and the cell body. Maximum deformation was measured in gel sub-regions (see Methods) by interpolating bead displacements onto a regular grid during events of protrusion and retraction. Independent protrusion and retraction events show no significant difference (P = 0.61 and P = 0.18, for FN and LN respectively, in a cell paired t-test), indicating that in each case we measured the maximum deformation of the gel. Cells plated on FN-coated gels deform them significantly more than LN-coated gels (P = 0.028 and P = 0.016 for protrusion and retractions respectively), suggesting that they exert stronger forces. Data are expressed as the mean ± SEM (n = 13 cells).

Together, our data suggest a weakened ligand-integrin-actin linkage in cells migrating using α6β1 or αLβ2 integrins. In this interpretation, the traction forces exerted by migrating cells on the substratum would be lower. To test this, we plated CHO.B2 cells expressing α5-GFP or α6-GFP on fluorescent bead embedded polyacrylamide gels coated with covalently-bound fibronectin or laminin, respectively [41], [42]. The maximum gel deformation was measured for protrusion and retraction events; however, the relative displacements for different events were similar on a given substrate (P = 0.61 and P = 0.18, for fibronectin and laminin respectively, in a cell paired t-test), indicating that in each case we measured the maximum deformation of the gel. Overall, the gel deformation during protrusions and retractions with α5-expressing cells was significantly higher than those in α6-expressing cells (Figure 7), suggesting that the α5-expressing cells generate higher traction on fibronectin than the α6-expressing cells do on laminin (P = 0.028 and P = 0.016 for protrusion and retractions respectively).

Reduced forces suggest that the signaling by adhesions might also be altered since adhesive signaling is thought to be force dependent [57], [8]. Therefore, we determined whether the differences in fluxing and traction force affected the signals produced by the adhesions in protrusions using a mGFP-dSH2 probe that binds to a Src-like kinase mediated phosphorylation of tyrosine on adhesion proteins [28], [12]. Cells migrating on fibronectin display small adhesions at the front of protrusions, and large adhesions at the rear, which result from myosin II activation [12]. The small adhesions exhibit high levels of mGFP-dSH2, indicating that they signal actively, whereas large adhesions display relatively low levels of mGFP-dSH2 [12]. Conversely, α6β1-expressing cells on laminin and αLβ2-expressing cells on ICAM-1 contain large adhesions even in their protrusions. They are comparable in size to those observed at the back of α5β1-expressing cells. However, these adhesions displayed an accumulation of the mCherry-dSH2 probe (Figure S5A). This indicates that large adhesions can signal robustly when cell adhesion occurs via α6β1or αLβ2, in contrast to adhesions of similar size forming in cells on fibronectin.

### Rapid Leukocyte Migration Arises from Cell Type Differences Rather than Alterations in Myosin II Activity or the Ligand-integrin-actin Linkage

**Figure 8:**
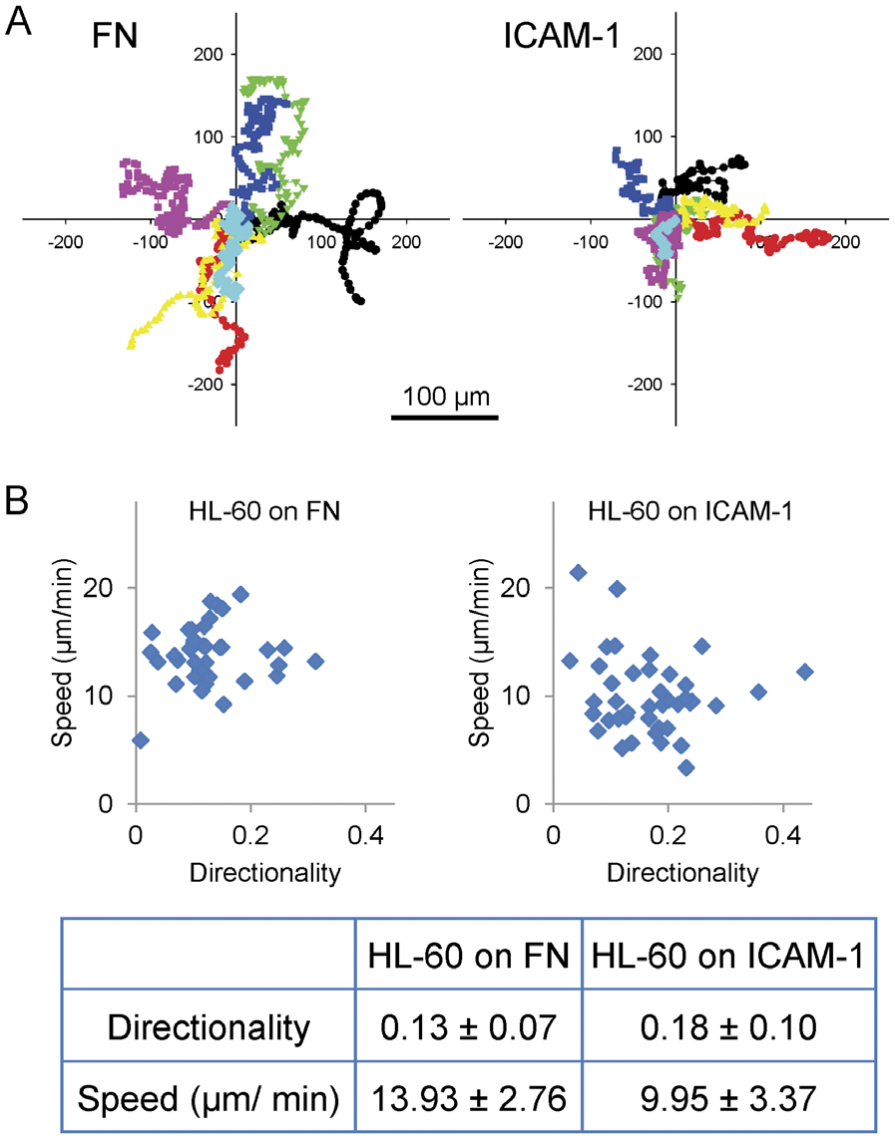
HL-60 migration on FN or ICAM-1. (A) Migration tracks of HL-60 cells on the indicated substrate over 1 hour were translated to a common origin and marked with a different color. Scale Bar  = 100 µm. (B) The speed and directionality were calculated and plotted (n = 36, 39 cell tracks, respectively). At least three independent experiments were quantified. P value  = 3×10^−7^.

We next sought to assess the contribution of cell type to the differences in adhesion and migration reported above in the CHO cells. We first compared the migration of CHO.B2 cells ectopically expressing αLβ2, with the spontaneous migration of promyelocytic leukemia (HL-60) cells, which become highly migratory following differentiation into neutrophil-like cells with DMSO [29]. HL-60 cells use αLβ2 to migrate on ICAM-1 and α4β1/α5β1 to migrate on fibronectin. When plated on fibronectin or ICAM-1, HL-60 cells migrate roughly 10 times faster than CHO.B2 cells expressing either α5 or αLβ2 and migrating on fibronectin or ICAM-1, respectively (Figure 8A and 8B). In addition, their adhesions are small in the periphery with a focal subventral area of contact with the substrate [58] (movie S2). This rapid migration is consistent with previous reports of high speeds and a polarized morphology consisting of a small leading protrusion [14] (Figure S5B).

**Figure 9:**
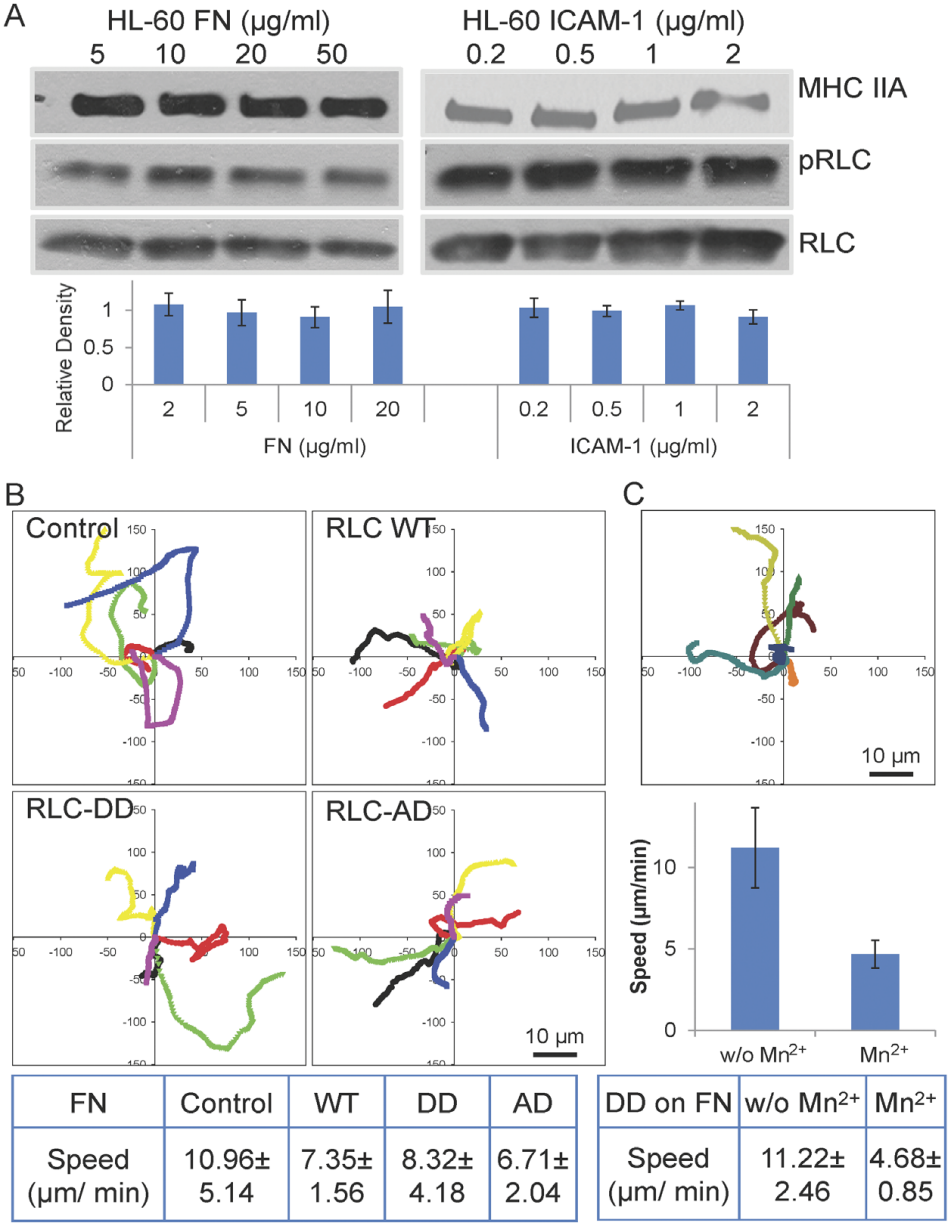
Effect of myosin II activation and Mn^2+^ on HL-60 cell migration. (A) Immunoblotting for pRLC shows that cells migrating on FN or ICAM-1 show similar levels. For the HL-60 cells, pRLC levels appear to be dose independent on both substrates. Representative blots are shown, and relative-fold increase of pRLC presented. The data are derived from at least three experiments for each substrate. (B) RLC activation does not affect the migratory properties of HL-60 cells on FN. HL-60 cells were doubly transfected with paxillin-mCherry and RLC, RLC-A,D, or RLC-D,D with GFP. Experimental time: 4 minutes. Scale Bar  = 10 µm. (C) Migration of HL-60 cells was inhibited by Mn^2+^. Three individual cells before and after Mn^2+^ treatment were plotted. Set 1: red; set 2: green; set 3: blue. P value  = 0.012.

We also analyzed the phosphorylation of RLC in response to adhesion. RLC phosphorylation is generally higher than that observed in CHO.B2 cells but largely ligand concentration-independent (Figure 9A). Adhesion and migration of the HL-60 cells were not significantly altered by the RLC mutants, RLC-A,D and RLC-D,D, regardless of whether the cells were migrating on fibronectin or ICAM-1(Figure 9B, data not shown). In addition, both RLC mutants localized to the rear of the cell as does wild type and endogenous RLC, as reported previously [59], [14]. Interestingly, the adhesions in the HL-60 cells, as visualized using paxillin-mCherry, were present as small dot-like structures at the side of cell, the uropod, and along the retraction path. The “touch, hold, and release” motion of the adhesions is clearly visible at the side edge and the rear of the cell, especially when the cell is changing direction (movie S2).

Therefore, HL-60 adhesion and migration differs from that of the CHO cells using the same integrin-ligand pairs and is largely refractory to increases in myosin II activity. Since their adhesions are small and do not appear to flux retrograde, it appears that the major difference between HL-60 and CHO cells resides in fundamental differences in cytoskeleton organization (e.g. actin organization) rather than to large differences in myosin II mediated contractile forces.

## Discussion

We have addressed the effects of different integrins on migration and adhesion using a common cell type expressing different integrins and different cell types expressing the same integrins. In contrast to cells migrating on fibronectin using the α5β1 integrin, the same cells migrating on laminin or ICAM-1 using the α6β1 and αLβ2 integrins, respectively, show the following phenotypes: increased migration rates and directional persistence, a rapid fluxing of integrins and other adhesion components in protrusions, a reduced effect of myosin II activation on migration, increased tyrosine phosphorylation in large adhesions, decreased adhesion strength, and inhibited force transmission from actomyosin to the substratum. In contrast, HL-60 cells, a leukocyte model, migrating on either fibronectin or ICAM-1 exhibit rapid migration and only small transient adhesions that do not flux and are largely independent of the myosin II activity. These observations indicate that intrinsic cellular differences, e.g., actin organization, regulate their adhesion and migration.

The striking differences in adhesion and migration between cells expressing α5β1 and α6β1 or αLβ2 and their decreased dependence on myosin II activity were unexpected. Both α5 and α6 dimerize with the β1 subunit in these cells and therefore potentially share similar signaling properties through the β1 cytoplasmic domain. While functional differences between α5β1 and α6β1 have been reported previously, their specific role in the signaling that controls cell migration is not understood [19], [60], [61]. One possibility is the tail of the alpha chain modulates signaling by the beta chain. It is also possible that unique binding proteins may modulate signaling triggered by a given integrin; for example, tetraspanins associate with α6β1 but not α5β1 [62].

Retrograde fluxing of adhesion components has been reported previously for cells using αvβ3 and α5β1 integrins and arises primarily from membrane resistance and actomyosin driven retrograde forces [9], [10]. Our studies show that α6β1 and αLβ2 expressing cells on laminin and ICAM-1, respectively, show an unusually prominent and rapid retrograde fluxing of adhesion components when protrusions pause, and a novel retrograde fluxing of the integrins, themselves, that is not commonly seen with α5β1 on fibronectin. This fluxing will inhibit the transfer of cellular forces to the substratum and therefore is likely responsible, at least in part, for the reduced forces sensed by the substratum. The enhanced fluxing, in turn, appears to arise from altered adhesion strength. Taken together, these differences indicate that differential mechanotransduction underlies the different migration properties. Several recent studies propose tension as a regulator of both adhesion maturation and the signals that adhesions produce [10], [63]–[65], [12]. In this regard, it is particularly interesting that CHO cells expressing α6β1 and αLβ2 have very large adhesions with prominent tyrosine phosphorylation in their protrusions, despite the reduced tension on the substratum. Presumably this reflects the residual cross-linking activity that sustains the large adhesions and actin bundles even in the presence of reduced force. Recently, Oakes, et al. also showed a lack of correlation between adhesion size and force [66].

Several reports document the retrograde translation of adhesions and the fluxing of molecules within them [54], [9], [52]. The translation appears to arise from the release of complexes at the rear of the adhesion and addition of new components in the front, i.e., direction of movement [67], [54], [27] presumably along actin filaments; although a net physical movement has not been ruled out. The retrograde movement of adhesion components presumably also arises from a treadmilling mechanism and reveals a clutch-like effect in which components closely associated with actin, like α-actinin, flux rapidly [9], [51]; whereas other components more closely associated with the substratum flux more slowly or not at all. Most studies have focused on the αvβ3 and α5β1 integrins in cells adhering to fibronectin. In these previous studies, the integrins are largely immobile suggesting that the force sensitive interaction lies in the associations among cytoplasmic components that comprise the integrin-actin linkage [9], [51]. Changes in the efficiency of the interaction can affect cell signaling by adhesions, since contractile forces couple to the substratum through this linkage. The fluxing of integrins that we observe for α6β1 and αLβ2 is novel and reveals a new force sensitive locus. The fluxing itself, inhibition by Mn^2+^ and the FRAP data suggest that the effective adhesion strength, e.g., apparent affinity, avidity or linkage to actin, is reduced.

The adhesion, migration, and polarity of fibroblast-like cells migrating on fibronectin depend on the activation status of myosin II via a poorly understood signaling loop [11], [21], [23], [12], [68], [69]. As RLC phosphorylation status increases, migration rates show a biphasic dependence, and small nascent adhesions in the lamellipodium tend to undergo rapid maturation into larger adhesions tethered to actin bundles. The adhesions in the protrusions show high relative phosphotyrosine levels; whereas larger adhesions outside the protrusions do not. Manipulating the levels of myosin II activity by knockdown, overexpression, inhibition, or altering RLC phosphorylation using mutants or RLC phosphatase or kinase inhibitors, all produce dramatic effects. In contrast, the migration of the same cells on laminin or ICAM-1 is more rapid, less dependent on either RLC phosphorylation or substrate density, and the adhesions in protrusions are large and highly elongated with high relative levels of phosphotyrosine. Based on fibroblast studies on fibronectin, large adhesions arise from the high tension generated by rigid substrata and tend to have reduced phosphotyrosine [12], [70]. However, laminin- or ICAM-1-based adhesions undergo retrograde flow and are under less tension. It is interesting in this context that the large adhesions in the α6β1 and αLβ2 expressing cells still contain tyrosine phosphorylated molecules. This reinforces the notion that force transmission through myosin II, rather than adhesion size, per se, regulates phosphotyrosine phosphorylation.

The difference in migration and response to RLC activation among different cell types using the same integrins is surprising. The CHO.B2 cells migrate faster on ICAM-1 or laminin than on fibronectin; yet HL-60 cells migrate faster on fibronectin, via α4β1 or α5β1, than on ICAM-1. In addition, the adhesions in the protrusions of migrating HL-60 cells are small despite the high level of RLC phosphorylation. Myosin IIB, which regulates polarity in CHO cell migration [23], [12], is not present in the HL-60 cells and provides a partial explanation for the differences in actin and myosin organization and their polarity. Dictyostelium also expresses a single myosin II [71] and shows migration properties closer to that of neutrophils [72]. Therefore, it appears that in more contractile cells, myosin II regulates the production of large actomyosin bundles and their associated adhesions. But for some highly migratory cells, the role of actomyosin is different and results in fundamentally different structures and associated adhesions. In these cells, the integrin-substrate linkage may largely serve as a brake at the back of the cell or while cells change direction, resulting in a more fluid movement due to the force provided by actin polymerization at the leading edge.

## Supporting Information

Figure S1 **Phase contrast images of CHO.B2 cells transfected with or without its appropriate integrins and plated onto FN, LN or ICAM-1. Cells do not spread without the necessary integrins.**
(TIF)

Figure S2 **Protrusion, RLC distribution and phosphorylation depend on integrin engagement in U2OS and HT1080 cells.** (A) Protrusion speed of HT-1080 (top) and U2OS (bottom) cells migrating on FN (1 µg/ml) or LN (5 µg/ml). Data are the mean ± SD of 17 independent measurements per condition. (B) Differential phosphorylation of RLC in response to increasing amounts of FN (left) and LN (right) in U2OS. Cells were plated for 60 min on either substrate, in the presence of RLC phosphorylation inhibitors (Y/M stands for 20 µM Y27632+10 µM ML7) or a phosphatase inhibitor (calyculinA, 10 nM), and blotted for phosphorylated (p) or total (t) RLC. Quantification (bottom) represents the mean ± SD of three independent experiments. (C) Subcellular distribution of the adhesion marker vinculin, actin and pRLC in U2OS plated on FN (1 µg/ml) or LN (5 µg/ml) for 60 min. Representative cells are shown. Scale Bar  = 10 µm.
(TIF)

Figure S3 (A) Inhibiting MII activity does not change protrusion rates. Upper panel: CHO.K1 cells, or CHO.B2 cells transfected cells with α6, were plated onto fibronectin or laminin, respectively, then treated with ML7, Y27632, both or Control (blebbistatin) for half hour. The level of pRLC was clearly reduced. Typical immunoblots of pRLC, with quantification of 3 blots, are shown. Lower panel: CHO.B2 cells transfected cells with integrin-GFP were plated on fibronectin or laminin and treated with ML7 or Y27632. Protrusion rates were calculated from kymographs. No significant difference was observed for cells on same substrate. (B) CHO.B2 cells expressing the appropriate GFP-coupled integrin were co-transfected with the indicated mCherry mutants, allowed to adhere to the corresponding substrate, and protrusion was assayed by kymography. Data are the mean ± SD of at least 3 independent experiments with 8–19 measurements per condition. There were no significant differences caused by expression of the RLC mutants.
(TIF)

Figure S4 (A) pRLC levels and protrusion rates on cells with different integrin expression levels. CHO.B2 cells were co-transfected with paxillin-mCherry and the appropriate integrin-GFP, sorted into three population by FACS: very low, low-medium, and high fluorescence, and then plated on FN or LN. Immunoblots for pRLC (upper panel) and protrusion rates are shown. High α5-GFP expressing cells show a small increase in pRLC level. Despite the integrin-GFP expression level, the protrusion rates remained similar on same substrate. (B) Relation between retrograde flow velocities, as determined by STICS, and average fluorescence protein expression level as estimated by ICS. Each point corresponds to a cell in a specific condition. Little, if any, influence of the expression level is observed on the retrograde fluxing. Correlation coefficients were computed for each condition (average of 0.24) and none was significant (P>>0.05).
(TIF)

Figure S5 (A) Adhesions in protrusions of CHO.B2 cells expressing α6-GFP or αL+β2-GFP express SH2 domain binding sites. CHO.B2 cells were double transfected with SH2-mCherry and α5-GFP, α6-GFP or αL+β2-GFP and plated on FN, LN or ICAM-1, respectively (Upper, middle or lower panel, respectively). For the merged channel, SH2-mCherry is in purple and integrins are in green. Scale Bar  = 10 µm. (B) T lymphocytes migrating on fibronectin display small, nascent adhesions. Jurkat E6.1 cells were transfected with GFP-vinculin, plated on FN (10 µg/ml) and allowed to migrate. The adhesions were imaged using TIRF microscopy after 30 min plating. Images were captured every 10 seconds for 1 hour. Representative time points are shown. Arrows indicate small adhesions that can be visualized as the cell extends new protrusions and retracts the rear. Scale Bar  = 5 µm.
(TIF)

Movie S1 CHO.B2 cells expressing different integrins show differential integrin engagement and paxillin and integrin dynamics. CHO.B2 cells expressing paxillin-mCherry and α5, α6-GFP or αL+β2-GFP (left, center, or right, respectively) were imaged using a 60X TIRF microscope as they migrate on FN, LN or ICAM-1, respectively. Paxillin-mCherry is in magenta and GFP-integrins are in green. Images were captured every 2 seconds, and the total imaging time was 4 minutes.
(AVI)

Movie S2 HL60 cells expressing paxillin-mCherry and migrating on FN (left and center) or ICAM-1 (right). Notice the touch, hold and release motion of paxillin (black dots and patches). Images were captured every 2 seconds and total imaging time was 4 minutes.
(AVI)
