# Supplementary figures and images for: The Integrin-Ligand Interaction Regulates Adhesion and Migration through a Molecular Clutch
Source: PLoS One. 2012 Jul 6;7(7):e40202. doi: 10.1371/journal.pone.0040202 (PMC3391238; doi:10.1371/journal.pone.0040202)

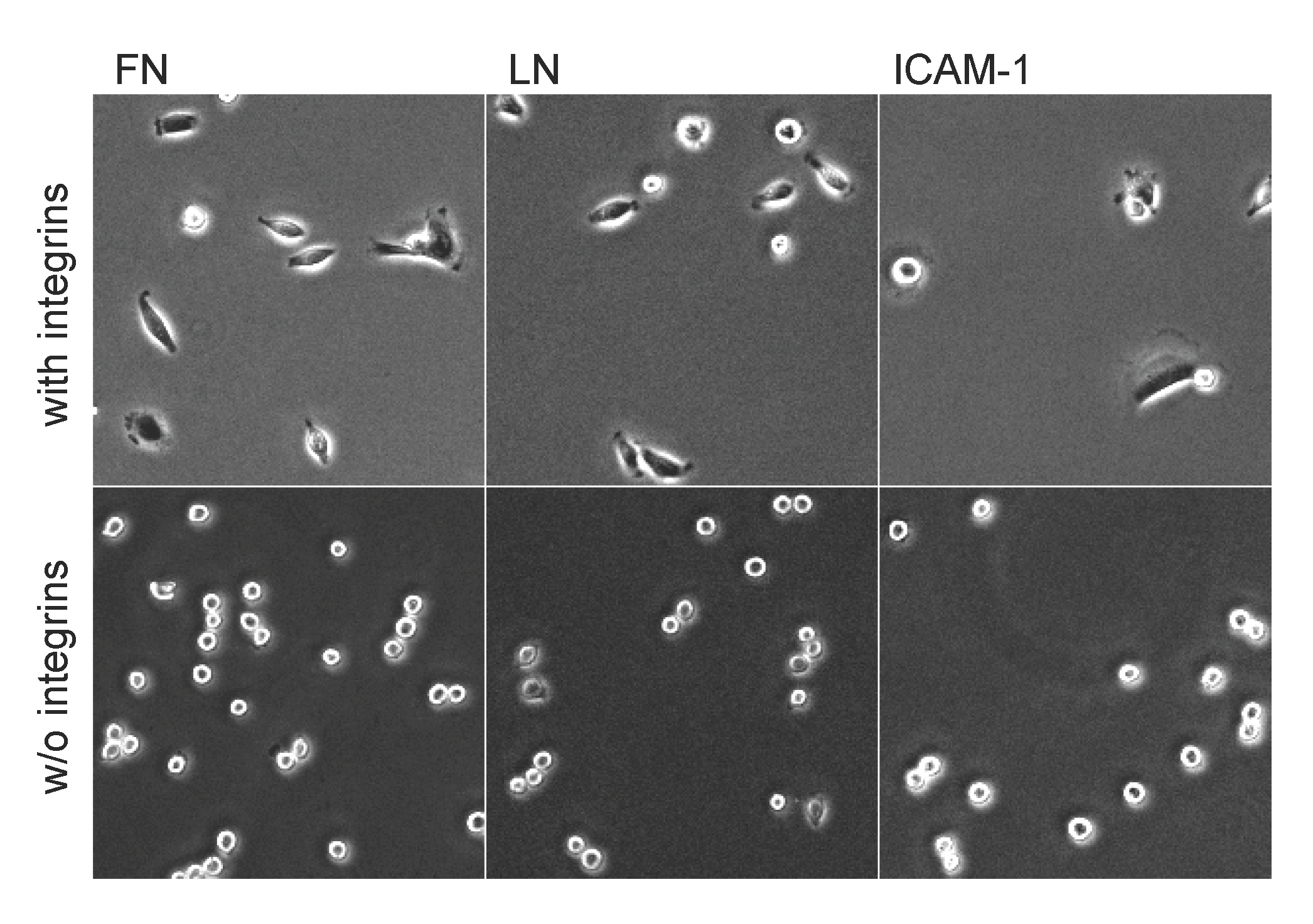

Supplement: Figure S1 — Phase contrast images of CHO.B2 cells transfected with or without its appropriate integrins and plated onto FN, LN or ICAM-1. Cells do not spread without the necessary integrins. (TIF) [file pone.0040202.s001.tif]

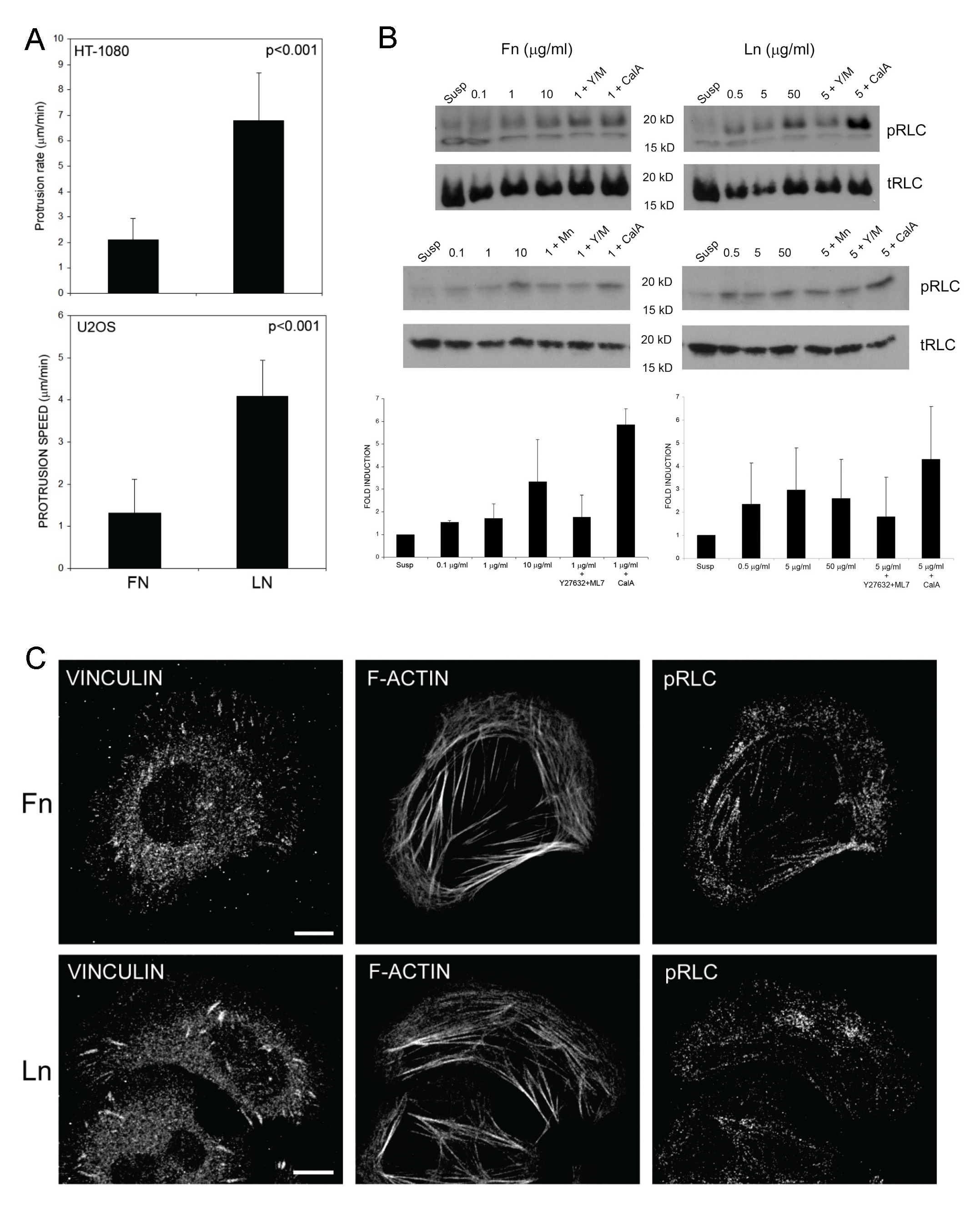

Supplement: Figure S2 — Protrusion, RLC distribution and phosphorylation depend on integrin engagement in U2OS and HT1080 cells. (A) Protrusion speed of HT-1080 (top) and U2OS (bottom) cells migrating on FN (1 µg/ml) or LN (5 µg/ml). Data are the mean ± SD of 17 independent measurements per condition. (B) Differential phosphorylation of RLC in response to increasing amounts of FN (left) and LN (right) in U2OS. Cells were plated for 60 min on either substrate, in the presence of RLC phosphorylation inhibitors (Y/M stands for 20 µM Y27632+10 µM ML7) or a phosphatase inhibitor (calyculinA, 10 nM), and blotted for phosphorylated (p) or total (t) RLC. Quantification (bottom) represents the mean ± SD of three independent experiments. (C) Subcellular distribution of the adhesion marker vinculin, actin and pRLC in U2OS plated on FN (1 µg/ml) or LN (5 µg/ml) for 60 min. Representative cells are shown. Scale Bar = 10 µm. (TIF) [file pone.0040202.s002.tif]

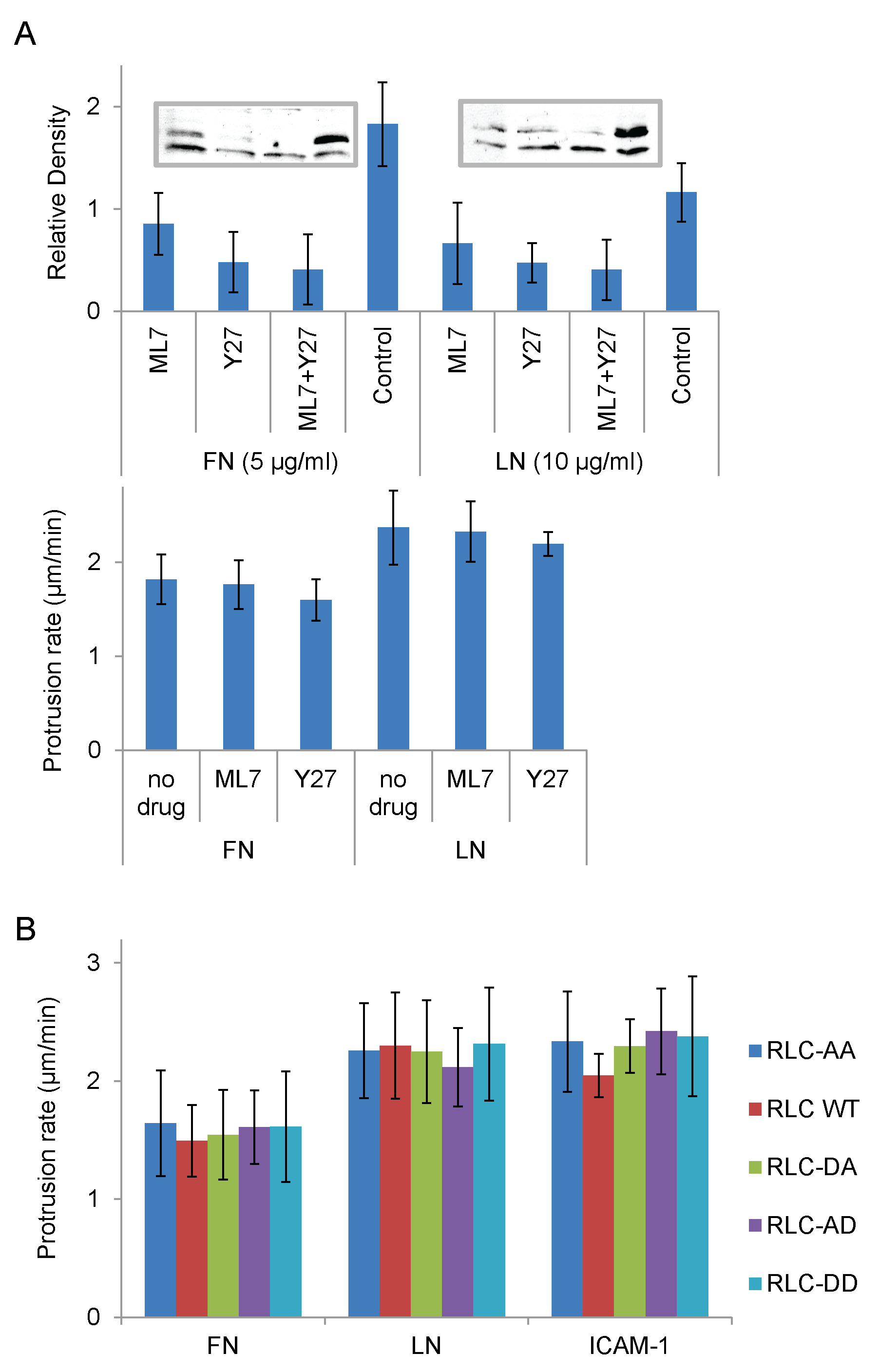

Supplement: Figure S3 — (A) Inhibiting MII activity does not change protrusion rates. Upper panel: CHO.K1 cells, or CHO.B2 cells transfected cells with α6, were plated onto fibronectin or laminin, respectively, then treated with ML7, Y27632, both or Control (blebbistatin) for half hour. The level of pRLC was clearly reduced. Typical immunoblots of pRLC, with quantification of 3 blots, are shown. Lower panel: CHO.B2 cells transfected cells with integrin-GFP were plated on fibronectin or laminin and treated with ML7 or Y27632. Protrusion rates were calculated from kymographs. No significant difference was observed for cells on same substrate. (B) CHO.B2 cells expressing the appropriate GFP-coupled integrin were co-transfected with the indicated mCherry mutants, allowed to adhere to the corresponding substrate, and protrusion was assayed by kymography. Data are the mean ± SD of at least 3 independent experiments with 8–19 measurements per condition. There were no significant differences caused by expression of the RLC mutants. (TIF) [file pone.0040202.s003.tif]

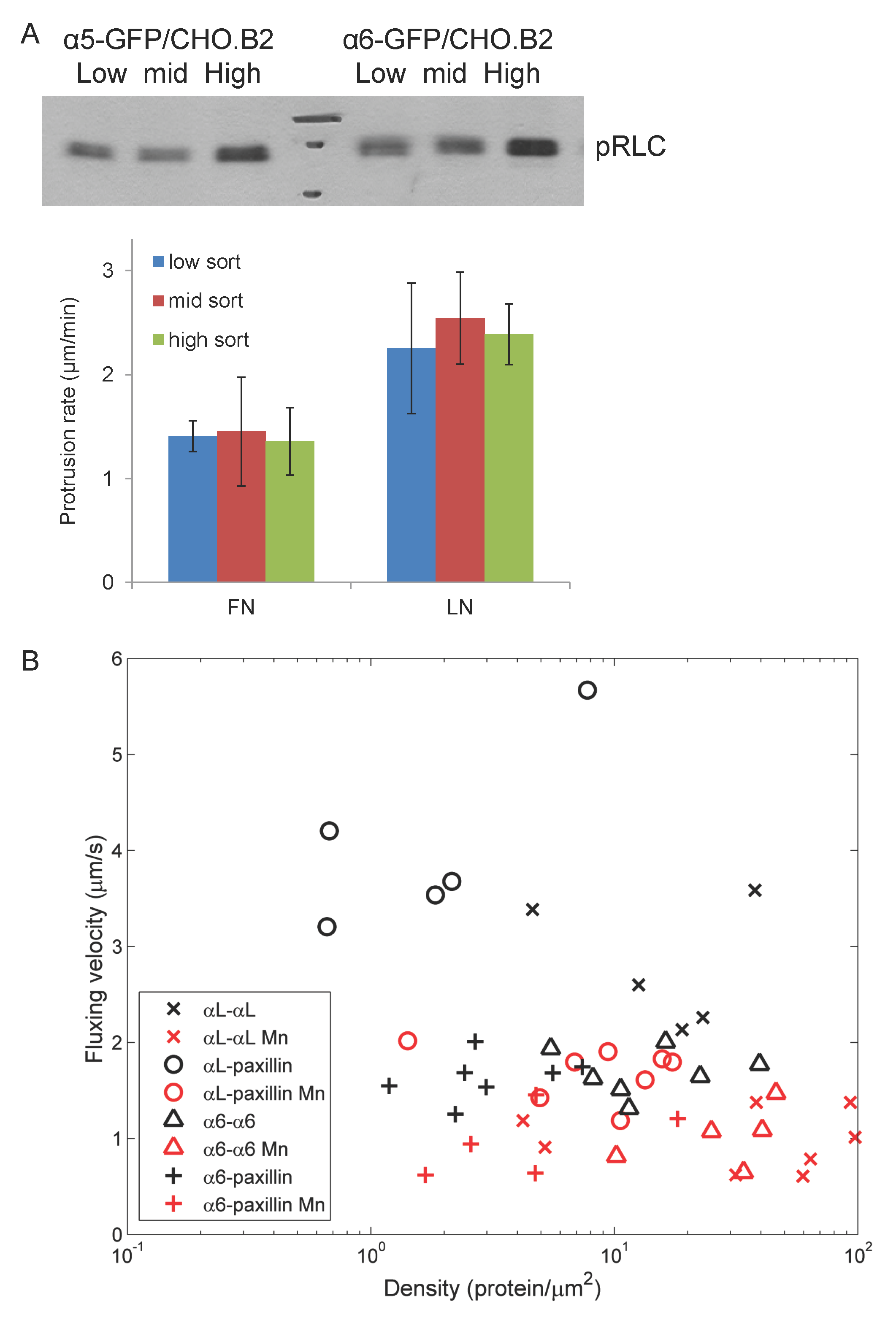

Supplement: Figure S4 — (A) pRLC levels and protrusion rates on cells with different integrin expression levels. CHO.B2 cells were co-transfected with paxillin-mCherry and the appropriate integrin-GFP, sorted into three population by FACS: very low, low-medium, and high fluorescence, and then plated on FN or LN. Immunoblots for pRLC (upper panel) and protrusion rates are shown. High α5-GFP expressing cells show a small increase in pRLC level. Despite the integrin-GFP expression level, the protrusion rates remained similar on same substrate. (B) Relation between retrograde flow velocities, as determined by STICS, and average fluorescence protein expression level as estimated by ICS. Each point corresponds to a cell in a specific condition. Little, if any, influence of the expression level is observed on the retrograde fluxing. Correlation coefficients were computed for each condition (average of 0.24) and none was significant (P>>0.05). (TIF) [file pone.0040202.s004.tif]

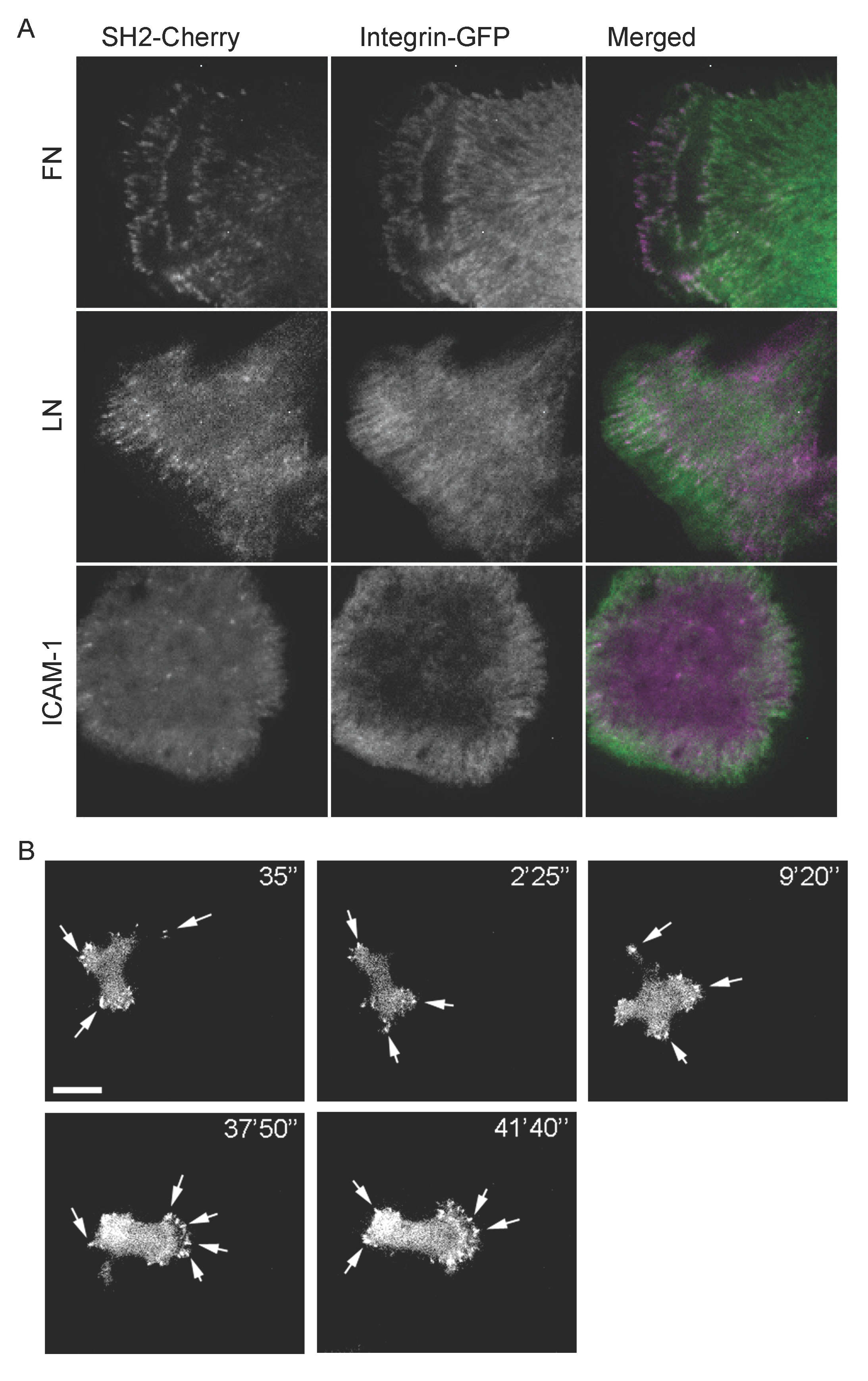

Supplement: Figure S5 — (A) Adhesions in protrusions of CHO.B2 cells expressing α6-GFP or αL+β2-GFP express SH2 domain binding sites. CHO.B2 cells were double transfected with SH2-mCherry and α5-GFP, α6-GFP or αL+β2-GFP and plated on FN, LN or ICAM-1, respectively (Upper, middle or lower panel, respectively). For the merged channel, SH2-mCherry is in purple and integrins are in green. Scale Bar = 10 µm. (B) T lymphocytes migrating on fibronectin display small, nascent adhesions. Jurkat E6.1 cells were transfected with GFP-vinculin, plated on FN (10 µg/ml) and allowed to migrate. The adhesions were imaged using TIRF microscopy after 30 min plating. Images were captured every 10 seconds for 1 hour. Representative time points are shown. Arrows indicate small adhesions that can be visualized as the cell extends new protrusions and retracts the rear. Scale Bar = 5 µm. (TIF) [file pone.0040202.s005.tif]
